# RE^3+^-Doped Ca_3_(Nb,Ga)_5_O_12_ and Ca_3_(Li,Nb,Ga)_5_O_12_ Crystals (RE = Sm, Dy, and Pr): A Review of Current Achievements

**DOI:** 10.3390/ma16010269

**Published:** 2022-12-27

**Authors:** Cristina Gheorghe, Stefania Hau, Lucian Gheorghe, Flavius Voicu, Madalin Greculeasa, Alin Broasca, George Stanciu

**Affiliations:** 1 National Institute for Laser, Plasma and Radiation Physics, 077125 Magurele, Romania; 2 Doctoral School of Physics, Faculty of Physics, University of Bucharest, 077125 Magurele, Romania

**Keywords:** garnet crystals, Sm^3+^, Dy^3+^, Pr^3+^, visible emission

## Abstract

Spectroscopic characteristics of RE^3+^ ions (RE = Sm, Dy, and Pr) doped in partially disordered Ca_3_Nb_1.6875_Ga_3.1875_O_12_-CNGG and Ca_3_Li_0.275_Nb_1.775_Ga_2.95_O_12_-CLNGG crystals are reviewed in detail to assess their prospects as laser crystals with emission in the visible spectral domain. All investigated crystals were grown using the Czochralski crystal growth technique. High-resolution absorption and emission measurements at different temperatures, as well as emission dynamics measurements, were performed on the grown crystals. The spectroscopic and laser emission characteristics of the obtained crystals were determined based on the Judd-Ofelt parameters. The obtained results indicate that CNGG:RE^3+^ and CLNGG:RE^3+^ (RE = Sm, Dy, and Pr) crystals can be promising materials for lasers in the visible range.

## 1. Introduction

A natural limitation of solid-state lasers due to the nature of the quantum processes underlying their operation is the existence of well-specified laser emission wavelengths determined by both the laser active ion and the host material. This situation requires further identification of new laser materials or emission schemes. One of the most viable solutions is to use host crystals with partially disordered structures determined by the mixed occupation of the host cationic sites with different ions. One of the most viable solutions is the use of host crystals with a partially disordered structure caused by the simultaneous occupation of some of the host cationic sites with different ions. Partial compositional disorder preserves the global crystallographic structure and symmetry of the crystal, but influences properties dependent on the local composition, such as the crystal field in the position of the laser active ion. Significant in this respect is the possibility of controlling the host disordering by producing manageable effects on the dopant ion spectra (especially the absorption and emission linewidths) through suitable control of the crystal field distribution in a compositionally disordered crystal. In the case of intrinsically disordered crystals in which certain crystallographic sites are filled with different types of cations with varying valences, the charge compensation induced by doping is achieved by changing the amounts of host cations. Due to the disorder in the close vicinity of the dopant ion, a multicenter structure and an inhomogeneous widening of the absorption and emission lines occur. Controlling the compositional disordering of the host laser material and a proper selection of the materials with an intrinsic disorder, are essential for identifying new laser materials or emission regimes.

Many families of disordered crystals such as aluminates, niobates, borates, double tungstates, etc., are currently known [1,2,3,4,5,6,7], but most of them are of low symmetry. Over recent years, trivalent rare earth ions (RE^3+^) doped in intrinsically disordered calcium-niobium-gallium-garnet (CNGG) and calcium-lithium-niobium-gallium-garnet (CLNGG) crystals have regained much interest as laser materials. Their main advantages are the low melting temperature (~1480 °C) [8,9] which allows the use of cheaper platinum (Pt) crucibles than iridium (Ir) ones, a moderate thermal conduction of 3–4.7 Wm^−1^ K^−1^, and the broad transition lines [9,10]. The stoichiometric composition of CNGG (Ca_3_Nb_1.5_Ga_3.5_O_12_) is different from the congruent one Ca_3_Nb_1.6875_Ga_3.1875_?_0.125_O_12_ which has a deficiency of Ga^3+^ and an excess of Nb^5+^ compensated by cationic vacancies (?) [8,9,11,12,13], which can be harmful, especially in the case of high-average power lasers. By adding Li^+^ cations into the CNGG crystal, the vacancies are eliminated and a new Ca_3_Li_y_Nb_(1.5+y)_Ga_(3.5–2y)_O_12_ (CLNGG) crystal is obtained. It was found that good quality CLNGG crystals can be obtained for the compositional parameter y comprised between 0.25 and 0.275, and the best results in terms of transparency of the grown crystal, the starting material purity, and the purity of the solidified melt remaining after crystal growth can be attained for y = 0.275 [9,10,12,14,15,16]. Regarding the cationic occupation in the CNGG structure, Ca^2+^ ions occupy dodecahedral *c*-sites, Nb^5+^, Ga^3+^, and vacancies (?) share the octahedral *a*-sites, and Ga^3+^ ions are situated in the tetrahedral *d*-sites. A small percentage (2% to a maximum of 5%) of the tetrahedral *d*-sites can be occupied by Nb^5+^ ions. In the CLNGG structure, Li^+^ ions are mainly located in the octahedral sites and less likely positioned in the tetrahedral sites [15]. In the CNGG and CLNGG structure, RE^3+^ ions replace Ca^2+^ cations in the dodecahedral positions of the garnet lattice, thus imposing the necessity of charge compensation by compositional modification of the host, mainly of the octahedral sublattices, according to each specific doping concentration. This determines the presence of cations of varying valence and/or ionic sizes in the first cation spheres of RE^3+^ ions, which further leads to the formation of a multicenter structure and an inhomogeneous broadening of the absorption and emission lines of RE^3+^ ions. The spectroscopic properties of different RE^3+^ (RE = Pr, Nd, Sm, Tb, Dy, Ho, Er, Tm, Yb) ions doped in CNGG or CLNGG crystals were reported [15,17,18,19,20,21,22,23,24,25]. For example, when doping with Nd^3+^ ions, five nearly similar nonequivalent centers were highlighted by low-temperature spectroscopic measurements on CNGG:Nd and CLNGG:Nd crystals [15]. Laser emission of Nd^3+^- or Yb^3+^-doped CNGG or CLNGG in various operating regimes and pumping conditions were reported [26,27,28,29,30,31,32].

The main criteria for the selection of Pr^3+^, Sm^3+^, and Dy^3+^ as doping ions are the electronic configuration and transition probabilities. Due to the strong absorption and emission transitions of the grouped manifolds, ^3^*P*_0_, ^3^*P*_1_, ^3^*P*_2_, and ^1^*I*_6_, respectively, the Pr^3+^ ion is a very attractive laser-active ion. The most important laser transitions of Pr^3+^ ions are in the red, yellow, orange, green, and blue spectral domains and arise from the ^3^*P*_0_ level [33,34,35,36,37]. Sm^3+^ ions exhibit strong orange-red luminescence assigned to the ^4^G_5/2_→^6^H_J_ (*J* = 5/2, 7/2, 9/2) electronic transitions and a long lifetime (ms) of the emission level, which make Sm^3+^ ion a perfect choice for phosphor materials. In recent years, due to the increased demand for different lasers and light sources in the visible (VIS) range, the research on Sm^3+^ ions for the development of yellow, red, or orange lasers under InGaN laser diode pumping has become more and more significant [21,38,39,40,41]. Similarly, Dy^3+^ ion is also a very good choice for investigating VIS emission. Yellow laser emission [42,43], white-light emitting phosphors [44,45], or blue emission for temperature sensing [46] were recently reported.

Optical properties of CNGG:RE^3+^ and CLNGG:RE^3+^ (RE = Sm, Dy, and Pr) crystals are systematically examined in this work to evaluate their prospects as laser crystals in the VIS domain. The oscillator strengths and transition probabilities of the 4*f*^2^, 4*f*^5^, and 4*f*^9^ electronic configurations of the trivalent Pr^3+^, Sm^3+^, and Dy^3+^ ions, respectively, were obtained. High-resolution spectroscopy at low (10 K) and room temperature was employed to determine the optical properties of RE^3+^ doping ions. The stimulated emission cross-sections were determined using the Füchtbauer-Ladenburg formula. The emission kinetics of the ^1^D_2_ (Pr^3+^), ^4^G_5/2_ (Sm^3+^), and ^4^F_9/2_ (Dy^3+^) excited levels were measured, and the Inokuti-Hirayama model was used to analyze the energy transfer processes and determine the transfer microparameters.

## 2. Materials and Methods

The polycrystalline host compounds Ca_3_Nb_1.6875_Ga_3.1875_O_12_ (CNGG) and Ca_3_Li_0.275_Nb_1.775_Ga_2.95_O_12_ (CLNGG) were synthesized by the solid-state reaction method. For doping of CNGG and CNLGG hosts with Sm^3+^, Dy^3+^, and Pr^3+^ ions, Sm_3_Ga_5_O_12_ (SmGG), Sm_3_Li_0.275_Nb_0.275_Ga_4.45_O_12_ (SmLNGG), Dy_3_Ga_5_O_12_ (DyGG), Dy_3_Li_0.275_Nb_0.275_Ga_4.45_O_12_ (DyLNGG), Pr_3_Ga_5_O_12_ (PrGG), and Pr_3_Li_0.275_Nb_0.275_Ga_4.45_O_12_ (PrLNGG) polycrystalline compounds, respectively, were also synthesized under the same conditions. The compositions of Li^+^-free compounds (SmGG, DyGG, PrGG) were selected according to the stoichiometric one, and the compositions of Li^+^-containing compounds (SmLNGG, DyLNGG, PrLNGG) were selected according to the optimal Li^+^ content of 0.275 in the CLNGG host. Powders with a purity of at least 4N of CaCO_3_, Nb_2_O_5_, Li_2_CO_3_, Ga_2_O_3_, Sm_2_O_3_, Dy_2_O_3_, and Pr_6_O_11_, were used as raw materials. In order to remove the absorbed water, CaCO_3_ and Li_2_CO_3_ powders were first heated at 400 °C for 10 h. Then, for each polycrystalline compound, all powders were weighed, mixed, pressed into cylindrical tablets, and heated for 12 h in an air atmosphere at 900 °C to decompose CaCO_3_ and Li_2_CO_3_. Finally, the tablets were further heated for 12 h at 1200 °C to complete the sintering. To achieve the 5 at.% doping level of the CNGG and CLNGG compounds with Sm^3+^, Dy^3+^, and Pr^3+^ ions, respectively, the polycrystalline compounds of the hosts and the corresponding dopants (SmGG, DyGG, PrGG in the case of CNGG, and SmLNGG, DyLNGG, PrLNGG for CLNGG) were mixed in the proper amounts, considering that the RE^3+^ (RE = Sm, Dy, and Pr) ions substitute Ca^2+^ in the CNGG and CLNGG hosts.

RE-doped CNGG (CNGG:RE) and RE-doped CLNGG (CLNGG:RE) crystals were grown using the Czochralski crystal growth technique from Pt crucibles with a height of 30 mm and a diameter of 30 mm in air atmosphere. The growth temperature was determined to lie between 1460 °C and 1480 °C. High-quality crystals of CNGG:RE and CLNGG:RE were grown along the <111> crystallographic direction using optimized rotation and pulling rates of 15–20 rpm and 1.3–1.5 mm/h, respectively. After growth, the crystals were thermally treated in an air atmosphere for 20 h at 1300 °C to release the thermal stress and stabilize the oxidation state of RE^3+^ doped ions. Detailed conditions about the growth parameters were presented in our earlier publication [47]. The chemical composition of the obtained crystals composition was determined by the inductively coupled plasma-atomic emission spectrometry (ICP−AES) method, and a Trace Scan Advantage spectrometer (Thermo Jarrell Ash Corp., Franklin, MA, USA) was used. The structural characterization of the sintered polycrystalline compounds and the grown crystals was done through X-ray powder diffraction (XRPD) using a Bruker AXS D8 ADVANCE X-ray diffractometer (Bruker, Brno, Czech Republic) with Cu Kα radiation (λ = 1.5406 Å). Rietveld analysis was employed to quantify any residual phases in the sintered compounds.

For low or room temperature spectroscopic measurements, a system containing a Jarrell Ash or Horiba Jobin Yvone monochromator (Jarrell Ash Division/Fisher Scientific Company, Waltham, MA, USA and Horiba Jobin Yvon GmbH, Bensheim, Germany, respectively) equipped with S20 or S1 type photomultipliers (Photek Ltd., St Leonards-on-Sea, East Sussex, UK) coupled to a lock-in amplifier coupled to a computer was employed. As excitation sources, a Melles Griot Argon laser (National Laser Comp., Salt Lake City, Utah, USA) or a tunable system containing an Oriel Cornerstone 260 monochromator (Newport Corporation, California, USA coupled with a monochromator illuminator Oriel APEX (Newport Corporation, California, USA) and a Xenon lamp (Sciencetech Inc., Ontario, Canada) were used. A closed cycle He refrigerator ARS-2HW (Advanced Research Systems, Inc., Macungie, PA, USA) was used for low-temperature measurements. To measure the luminescence decay, a Tektronix 2024B oscilloscope (Tektronix, Inc., Beaverton, OR, USA) was used for data recording, and a tunable OPOTEK RADIANT 355 LD laser (Opotek LLC., California, CA, USA) in the range of 410–2500 nm or the third harmonic at 355 nm of an Nd:YAG Quantel laser was utilized as an excitation source.

## 3. Results

### 3.1. Crystals Growth and Structural Characterization

XRPD spectra on the SmGG, DyGG, PrGG, SmLNGG, DyLNGG, and PrLNGG sintered compounds revealed a dominant garnet-type cubic phase (space group *Ia3d*) and some residual phases like Ga_2_O_3_, SmNbO_4_, LiGa_5_O_8_, DyNbO_4_, and PrGaO_3_. According to Rietveld analysis, the majority garnet phase was found to be in the range of 85–91 wt.%, while the total of minor secondary phases was quantified to be less than 15 wt.% in each sintered compound [47]. The relationships between interplanar spacings and Miller indices, as well as the lattice constants for the garnet phase in each sintered compound, are presented in Reference [47].

**Figure 1:**
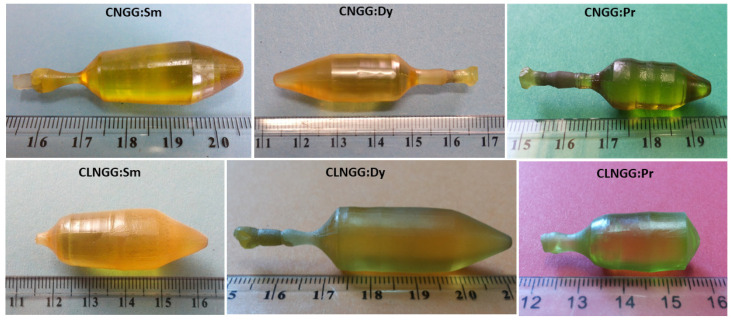
CNGG:RE and CLNGG:RE (RE = Sm, Dy, and Pr) obtained crystals. (Reprinted with permission from Ref. [47], copyright 2018, Elsevier).

Figure 1 shows the CNGG:RE and CLNGG:RE grown crystals. As can be observed, they have very good transparency, are free of macroscopic defects, and are of high optical quality. The grown crystal sizes are approximately 12 and 25–30 mm in diameter and length, respectively. The grown crystals present good mechanical properties, being hard enough for cutting and polishing.

**Figure 2:**
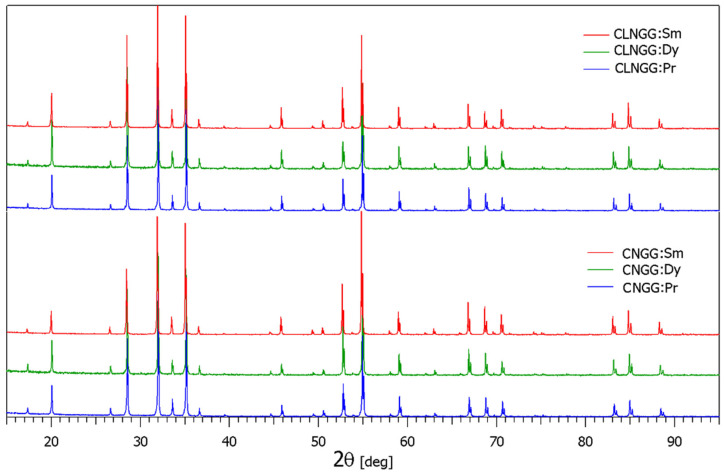
XRPD patterns of CNGG:RE and CLNGG:RE (RE = Sm, Dy, Pr) grown crystals. (Reprinted with permission from Ref. [47], copyright 2018, Elsevier).

The XRPD patterns on the CNGG:RE and CLNGG:RE grown crystals are shown in Figure 2. The patterns are very well indexed by the garnet-type structure (space group *Ia3d*) and no residual phases could be found. The lattice parameters were determined and are given in Table 1 together with those of the undoped host. The determined lattice parameters match very well with the ionic radii of Ca^2+^ (1.12 Å), Pr^3+^ (1.126 Å), Sm^3+^ (1.079 Å), and Dy^3+^ (1.027 Å) ions in 8-fold oxygen coordination, thus proving the insertion of RE^3+^ ions into the dodecahedral *c*-sites of the obtained crystals. Moreover, the elemental compositions of the crystals with a measurement error of ± 0.2% are given in Table 1. The effective segregation coefficients (k_eff_) of RE^3+^ ions in the CNGG host crystal were evaluated to be k_eff_ (Sm) = 0.69, k_eff_ (Dy) = 0.84, and k_eff_ (Pr) = 0.36, being similar to those obtained in the case of the CLNGG host crystal. Thus, the concentration of RE^3+^ dopant ions in CNGG:RE and CLNGG:RE crystals was determined as being 3.4 at.%, 4.2 at.%, and 1.8 at.% for Sm^3+^, Dy^3+^, and Pr^3+^, respectively. The cation densities of RE^3+^ ions were calculated to be *N_A_* = 4.236 × 10^20^ ions/cm^3^ (CNGG:Sm), *N_A_* = 5.14 × 10^20^ ions/cm^3^ (CNGG:Dy), and *N_A_* = 2.209 × 10^20^ ions/cm^3^ (CNGG:Pr) in the case of CNGG:RE crystals, and *N_A_* = 4.232 × 10^20^ ions/cm^3^ (CLNGG:Sm), *N_A_* = 5.15 × 10^20^ ions/cm^3^ (CLNGG:Dy), and *N_A_* = 2.204 × 10^20^ ions/cm^3^ (CLNGG:Pr) for CLNGG:RE crystals.

### 3.2. Spectroscopic Investigations

The room temperature absorption spectra of CNGG:RE and CLNGG:RE crystals (RE^3+^ = Pr^3+^, Sm^3+^, Dy^3+^) were registered and analyzed within the Judd-Ofelt (JO) theory [48,49] to calculate the oscillator strengths and transition probabilities of the 4*f*^2^, 4*f*^5^, and 4*f*^9^ electronic configurations of the Pr^3+^, Sm^3+^, and Dy^3+^ ions, respectively. The JO theory is described in detail in References [21,23,24], and the most important formulas are given below. The electric dipole line strength (*S_meas_*) of a transition can be determined from the absorption measurements as:

(1) $$S_{meas}\left(J\to J^{\prime }\right)=\frac{3ch\left(2J+1\right)n}{N_{A}3\pi^{3}\bar{\lambda }}\left[\frac{9}{{\left(n^{2}+2\right)}^{2}}\right]\int k\left(\lambda \right)d\lambda$$

where *c* is the speed of light, *h* is the Planck constant, *n* is the bulk index of refraction, *N_A_* is the RE^3+^ ion concentration, $\bar{\lambda }$ is the mean wavelength of the absorption band that corresponds to the *J→J’* transition, $\int k\left(\lambda \right)d\lambda$ is the integrated absorption coefficient, and *k*(λ) is the absorption coefficient which depends on the wavelength. Based on several values of the refractive indices at various wavelengths of undoped CNGG [50,51] and CLNGG [17] crystals and a least-squares fitting program for the Sellmeier equations, dispersion curves of refractive indices were determinate and further used to calculate *S_meas_* for each transition. The theoretical (*S_theor_*) [52] and measured (*S_meas_*) oscillator strengths of the absorption transitions were obtained and used to determine the *Ω_t_ (t = 2, 4, 6)* intensity parameters. The root-mean-square (rms) deviation $\;\Delta S_{rms}={\left[{\left(q-p\right)}^{-1}\sum {\left(\Delta S\right)}^{2}\right]}^{1/2}$, where *q* is the number of the analyzed transitions, *p* is the number of parameters, and *∆S = S_theor_-S_meas_*, represents the matching error.

Other spectroscopic parameters such as spontaneous emission probabilities (*A_JJ_*_’_), branching ratios (*β*), and radiative lifetimes (*τ_r_*), were determined based on JO intensity parameters. The total spontaneous electric dipole emission transition probabilities from the excited state *J* to the lower state *J’* are given by the formula:

(2) $$A_{JJ'}^{ed}=\frac{64\pi^{4}e^{3}}{3h\left(2J+1\right){\bar{\lambda }}^{3}}\frac{n{\left(n^{2}+2\right)}^{2}}{9}\underset{t=2,4,6}{\sum }\Omega_{t}\left|\left(S,L\right)J\right.U^{t}\left(S^{\prime },L^{\prime }\right){\left.J^{\prime }\right|}^{2}$$

The radiative lifetime *τ_r_* for an excited state *J* and the luminescence branching ratios *β(J→J’)* for the various emission transitions from this state can be then calculated as:

$$\tau_{r}=\frac{1}{\sum A\left(J\to J^{\prime }\right)},\;\mathrm{and}\;\beta \left(J\to J^{\prime }\right)=\frac{A\left(J\to J^{\prime }\right)}{\sum_{J^{\prime }}A\left(J\to J^{\prime }\right)}$$

From room temperature emission spectra originating from ^1^D_2_ (Pr^3+^), ^4^G_5/2_ (Sm^3+^), and ^4^F_9/2_ (Dy^3+^) manifolds, stimulated emission cross-sections *(σ_em_)* were calculated by using the Fuchtbauer-Ladenburg (FL) equation [53]:

(3) $$\sigma \left(\lambda \right)=\frac{\lambda_{}^{5}A\left(J\to J'\right)}{8\pi n^{2}c}\frac{I\left(\lambda \right)}{\int \lambda I\left(\lambda \right)d\lambda }$$

where *A (J→J’)* is the spontaneous emission probability from the excited state *J* to the terminal state *J’*, *I(λ)* is the emission intensity at wavelength *λ*, *n* is the refraction index, *c* is the speed of light, and $\int \lambda I\left(\lambda \right)d\lambda$ is the integrated emission intensity.

The energy transfer (ET) processes induced by the static interactions between the dopant ions strongly influence the excitation flow between the energy levels of the active ion. The interionic process represents the direct transfer of excitation between two ions without the absorption or emission of photons. The ions involved (donors D and acceptors) are connected through the multipolar, exchange, or super-exchange interactions explained by the theory developed by Förster [54] and Dexter [55]. The ET from the donor to the acceptor, in addition to radiative and non-radiative de-excitation, represents a process of de-excitation of the donor. This transfer modifies the excited level lifetime of the donor. Depending on the dopant concentration, the kinetics of the emission level can be described by an exponential or non-exponential function. At very low dopant concentration, the measured lifetime is likely to be the radiative lifetime if there is no non-radiative contribution to the decay curve. For higher dopant concentration, the non-exponential luminescence decays can be evaluated using the formula *τ_av_* =$\int_{0}^{\infty }tI\left(t\right)dt$/$\int_{0}^{\infty }I\left(t\right)dt$ and extracting an average lifetime for the emitting level. At high doping concentrations, the type of interaction between RE^3+^ ions, such as dipole-dipole (DD), dipole-quadrupole (DQ), or quadrupole-quadrupole (QQ) interaction, can be determined from the non-exponential profile of the luminescence decay. In this case, the Inokuti-Hirayama (IH) energy transfer model [56] was employed to analyze the emission decay by using the following equation for the luminescence intensity, *Φ(t)*:

(4) $$\Phi \left(t\right)=A\times \exp\left[-\frac{t}{\tau_{0}}-\frac{4\pi }{3}\Gamma \left(1-\frac{3}{s}\right)N_{A}R_{0}^{3}{\left(\frac{t}{\tau_{0}}\right)}^{\frac{3}{s}}\right]$$

where *A* is the amplitude, *τ_0_* is the lifetime of isolated RE^3+^ ions, *Γ* is Eulers’ function (*Γ* is 1.77, 1.43, and 1.3 for *s* = 6, 8, and 10, respectively), *s* is the mechanism of multipolar interaction (6 for DD, 8 for DQ, 10 for QQ), *N_A_* is the concentration of RE^3+^ ions, and *R_0_* is the critical transfer distance between two neighboring RE^3+^ ions. When non-radiative losses through cross-relaxation processes between two neighboring dopant ions are present, the microparameter of donor-acceptor interaction (*C_DA_*) and the transfer rates (*W_DA_*) can be calculated by the following equations, *C_DA_* = $R_{0}^{s}\tau_{0}^{-1}$ and *W_DA_ = C_DA_*/$R_{0}^{s}$, respectively. The energy transfer rate (*W_ET_*) through cross relaxation [57], as well as quantum efficiency *ɳ*, can be also calculated by the formulas WET=1τ−1τrad and *ɳ (ɳ = τ/τ_rad_*), respectively.

- Sm^3+^ ions

**Figure 3:**
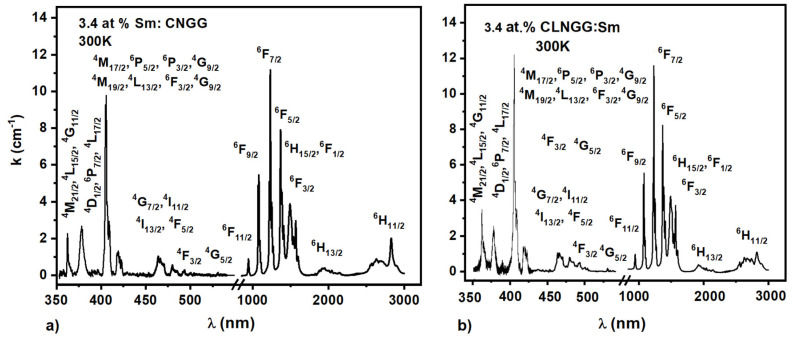
Absorption spectra at room temperature of Sm^3+^ ions in CNGG:Sm (**a**) and CLNGG:Sm (**b**) crystals.

The absorption spectra of 3.4 at.% Sm^3+^ ions in CNGG:Sm and CLNGG:Sm crystals were measured at 300 K in the spectral range of 350–3000 nm and analyzed within the JO theory [48,49]. The obtained spectra are shown in Figure 3. Twelve absorption lines of Sm^3+^ ions were identified and analyzed to determine the JO parameters [21].

Based on measured and calculated line strengths [21], the *Ω_t_* (*t = 2, 4, 6*) parameters of CNGG:Sm and CLNGG:Sm crystals were obtained and are listed in Table 2. The order *Ω_4_ > Ω_2_ > Ω_6_* of the parameters is in trend with that obtained for different Sm^3+^-doped crystals with similar structures [57,58,59,60]. The significance of each JO parameter was studied [61,62,63] and it was established that *Ω*_2_ is an intensity parameter very sensitive to the crystal field asymmetry in the RE^3+^ ion site, the covalency of the RE^3+^ ion, as well as to any modification in the energy gap between the 4*f^n^* and 4*f^n−1^5d* states of the RE^3+^ ion. *Ω*_6_ is a parameter that reacts more to any variation in the electron density of the 4*f* and 5*d* configurations. Any alteration of the *Ω_2_* and *Ω_6_* parameters have an impact on the *Ω_4_* parameter, which frequently complicates the establishment of the real factors that influence its modification. The values of spontaneous emission probabilities *(A_JJ’_)*, branching ratios *(β_JJ’_)*, and radiative lifetimes (*τ_r_*) for the ^4^G_5/2_ excited level were determined based on the *Ω_t_* (*t = 2, 4, 6*) parameters [21], and are given in Table 3. The radiative lifetime values of the ^4^G_5/2_ level were found to be 1.58 ms and 1.5 ms, for CNGG:Sm and CLNGG:Sm crystals, respectively.

**Figure 4:**
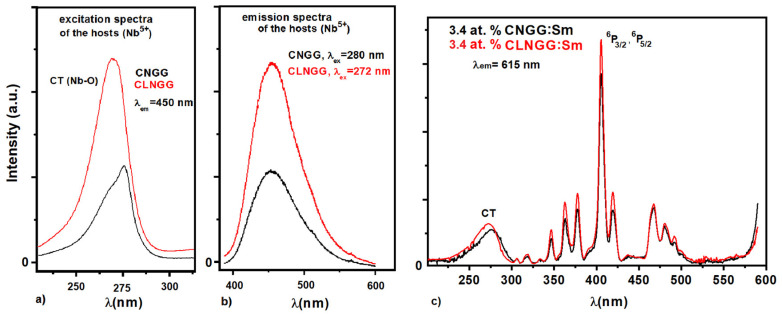
Excitation (**a**) and emission (**b**) spectra of the hosts, and excitation spectra of Sm^3+^ ions in CNGG:Sm and CLNGG:Sm crystals (**c**).

Figure 4a shows the excitation spectra of CNGG and CNLGG host crystals obtained by observing the blue emission line at 450 nm (Nb^5+^). The spectra exhibit a charge transfer (CT) band in the UV range positioned at 272 nm and 280 nm for CLNGG and CNGG, respectively, assigned to the Nb^5+^-O^2−^ interactions into the [NbO_4_] tetrahedrons of the host lattices [17]. Compared to CNGG, the shift to the higher energy of the CT band peak for CLNGG is due to the insertion of Li^+^ ions into the CNGG structure which practically removes the vacancies producing a slight distortion of the [NbO_4_] tetrahedrons. The emission spectra under 280 nm and 272 nm excitation wavelengths of the CNGG and CLNGG hosts, respectively, are shown in Figure 4b. As can be seen, both crystals display a wide emission band in the VIS range with a peak located at about 450 nm. Figure 4c shows the excitation spectra of Sm^3+^ ions in CNGG:Sm and CLNGG:Sm crystals by observing the emission line of Sm^3+^ ions in orange at 615 nm attributed to the ^4^G_5/2_→^6^H_7/2_ transition. The spectra present the CT bands from 200 to 300 nm assigned to the Nb^5+^-O^2−^ interactions into the [NbO_4_] tetrahedrons of the host lattices, and a group of narrow lines in the UV-VIS region assigned to the 4*f*^5^ electronic configuration of Sm^3+^ ions.

**Figure 5:**
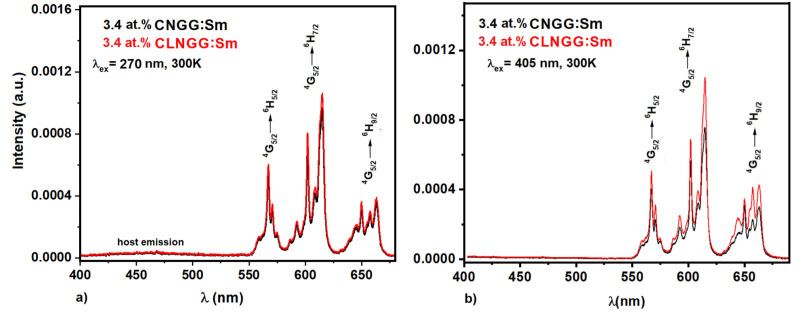
Emission spectra of Sm^3+^ ions in CNGG:Sm and CLNGG:Sm crystals under 270 nm (**a**) and 405 nm (**b**) excitation wavelengths.

The emission spectra of Sm^3+^ ions in CNGG:Sm and CLNGG:Sm crystals under 270 nm and 405 nm excitations were recorded at room temperature (Figure 5). Sm^3+^ ion has an intense absorption band around 405 nm attributed to the ^6^H_5/2_ → ^6^P_3/2_, ^6^P_5/2_ transitions, which is very appropriate for the efficient pumping with InGaN laser diodes at ~405 nm. The values of the absorption cross-sections for the peaks at 404.9 nm and 405.6 nm were determined to be 2.1 × 10^−20^ cm^2^ and 2.3 × 10^−20^ cm^2^, respectively, for CNGG:Sm, and 2.2 × 10^−20^ cm^2^ and 2.8 × 10^−20^ cm^2^, respectively, for the CLNGG:Sm crystal [21]. Under both excitation wavelengths (270 nm and 405 nm), the emission spectra of Sm^3+^ ions exhibit three emission bands centered at 567 nm, 615 nm, and 662 nm corresponding to the ^4^G_5/2_ → ^6^H_5/2_, ^4^G_5/2_ → ^6^H_7/2_, and ^4^G_5/2_ → ^6^H_9/2_ transitions, respectively. Under 270 nm excitation, a low-intensity emission band around 450 nm attributed to the host emission can be observed (Figure 5a). The emission line at 615 nm assigned to the ^4^G_5/2_ → ^6^H_7/2_ transition is the most intense line observed for both CNGG:Sm and CLNGG:Sm crystals, under both 270 nm and 405 nm excitation wavelengths. The emission cross sections were calculated for each emission band corresponding to the transitions from the ^4^G_5/2_ level to ^6^H_5/2_, ^6^H_7/2_, and ^6^H_9/2_ lower levels of Sm^3+^ ions in CNGG:Sm and CLNGG:Sm crystals. The highest values of the emission cross-sections were obtained for the CLNGG:Sm crystal at 615 nm (^4^G_5/2_→^6^H_7/2_) and 662 nm (^4^G_5/2_→^6^H_9/2_). All obtained values are presented and compared with other similar materials in Table 4. The values of Sm^3+^ emission cross-sections at 615 nm suggest that CNGG:Sm and CLNGG:Sm are potential laser crystals with orange emission at 615 nm.

The partial energy levels and the multiplet barycenter (*B_c_*) of Sm^3+^ ions in CNGG:Sm and CLNGG:Sm crystals were identified based on absorption and emission spectra at 10 K [21]. The energy levels involved in the main Sm^3+^ emission transitions are given and compared with those for the YAG:Sm crystal [57] in Table 5, to highlight the effect of partial structural disorder on the energy levels of Sm^3+^ in CNGG:Sm and CLNGG:Sm compared to the structurally ordered YAG:Sm crystal. The main crystal field effects are given by the perturbations of the crystal field induced by the disorder through the mixed occupation of the cationic sublattices, charge difference, and size mismatch effects. All these facts lead to the formation of multiple Sm^3+^ centers, a low local crystal field, as well as changes in the spectral properties of the Sm^3+^ ions by modifying the emission wavelengths.

**Figure 6:**
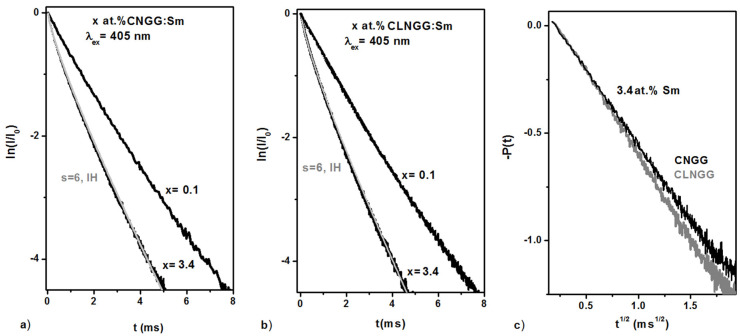
Emission kinetics of the ^4^G_5/2_ level of Sm^3+^ ions in CNGG:Sm (**a**) and CLNGG:Sm (**b**), and the experimental transfer function (**c**).

The luminescence kinetics of the ^4^G_5/2_ level of Sm^3+^ ions in CNGG:Sm and CLNGG:Sm at room temperature were recorded by observing the emission line at 615 nm under 405 nm excitation. To find out the lifetime of isolated Sm^3+^ ions, CNGG:Sm and CLNGG:Sm sintered ceramics doped with 0.1 at.% Sm^3+^ ions were also measured. The normalized decay curves are shown in Figure 6a,b. For 0.1 at. % Sm, the decay curves are nearly exponential, and the measured lifetimes were determined to be τ_0_ = 1.483 ms for CNGG:Sm and τ_0_ = 1.44 ms for CLNGG:Sm. The values obtained for isolated Sm^3+^ ions (*τ_0_*) are near to the values determined by the JO method, as being τ_r_ = 1.58 ms for the CNGG:Sm crystal and τ_r_ = 1.5 ms for the CLNGG:Sm crystal. This fact indicates that the values of *τ_0_* can be considered as the radiative lifetimes, since that the energy gap between the ^4^G_5/2_ level and the next lower level is about 7200 cm^−1^ and the maximum phonon energy in the hosts is about 750 cm^−1^ [12], thus resulting in a negligible non-radiative contribution to the luminescence decay.

For 3.4 at.% Sm, the decay curves present a non-exponential shape. The average luminescence lifetimes were estimated to be τ*_av_*=1 ms and τ*_av_* = 0.95 ms for CNGG:Sm and CLNGG:Sm crystals, respectively. Employing the Inokuti-Hirayama (IH) model, the non-exponential decay curves were evaluated, and the experimental transfer functions were determined (Figure 6c). From the fitting of the ^4^G_5/2_ decay curve, the critical distance (*R_0_*), microparameter of donor-acceptor interaction (*C_DA_*), transfer rates *(W_DA_),* energy transfer rate (*W_ET_*), and quantum efficiency (*ɳ*) were determined (Table 6).

The emission quenching of the ^4^G_5/2_ level with Sm concentration could be mostly due to the energy transfer (ET) by cross-relaxation [57]. The ET rate (*W_ET_*) is 325 s^−1^ and 358 s^−1^ for the CNGG:Sm and CLNGG:Sm crystals, respectively. The quantum efficiency *ɳ* of the ^4^G_5/2_ level, which is defined as the ratio of the number of photons emitted to the number of photons absorbed, was estimated to be ~66 % for both crystals. This shows that the multiphonon relaxations and the ET processes of the ^4^G_5/2_ level are negligible, which is advantageous for lasers and photonic devices.

- Dy^3+^ ions

**Figure 7:**
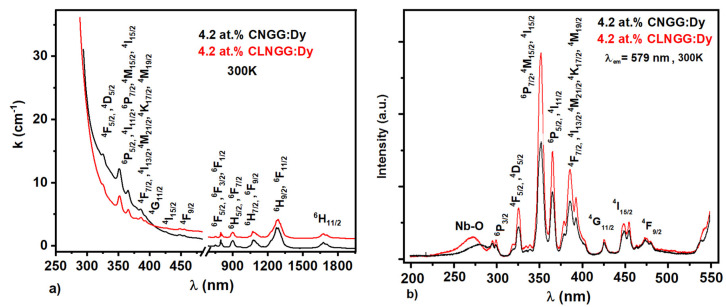
Absorption (**a**) and excitation (**b**) spectra of Dy^3+^ ions in CNGG:Dy and CLNGG:Dy crystals. (Reprinted with permission from Ref. [23], copyright 2018, Elsevier).

The absorption spectra of 4.2 at.% Dy^3+^ ions in CNGG:Dy and CLNGG:Dy crystals were recorded in the extended spectral domain of 330–2000 nm at room temperature (Figure 7a) [23]. The absorption spectra were analyzed within the JO theory [48,49] to evaluate the intensity parameters *Ω_t_* (*t = 2, 4, 6*) contributing to the determination of the electric and magnetic dipole spontaneous emission probabilities *(A_JJ’_)*, spectroscopic quality factor (*χ = Ω_4_/Ω_6_*), branching ratios *(β_JJ’_)*, and the radiative lifetime *(τ_r_)* of the ^4^F_9/2_ Dy^3+^ emitting manifold [23]. Table 7 summarizes all the results obtained.

Figure 7b shows the excitation spectra of Dy^3+^ ions in CNGG:Dy and CLNGG:Dy crystals [23] obtained by observing the yellow line at 579 nm assigned to the ^4^F_9/2_→^6^H_13/2_ transition. For both crystals, the spectra present a CT band in the UV range of 200–300 nm assigned to the Nb^5+^-O^2−^ interactions into the [NbO_4_] tetrahedrons of the host lattices, and a group of narrow lines in the UV-VIS domain assigned to the 4*f*^9^ electronic configuration of Dy^3+^ ions. Being positioned at wavelengths shorter than 200 nm according to Reference [68], the CT band due to the Dy^3+^−O^2−^ interaction could not be highlighted.

**Figure 8:**
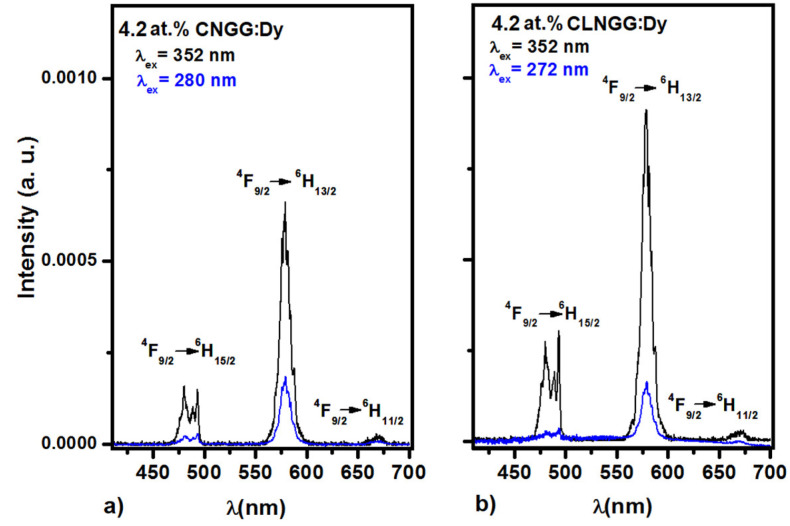
Emission spectra of Dy^3+^ ions in CNGG:Dy (**a**) and CLNGG:Dy (**b**) crystals at room temperature. (Reprinted with permission from Ref. [23], copyright 2018, Elsevier).

The emission spectra of Dy^3+^ ions in CNGG:Dy and CLNGG:Dy crystals (Figure 8a,b) were recorded at 300 K under different excitation wavelengths of λ_ex_ = 272, 280, and 352 nm. The obtained spectra show emission bands in the blue, yellow, and red spectral regions, assigned to the ^4^F_9/2_ → ^6^H_15/2_, ^4^F_9/2_ → ^6^H_13/2_, and ^4^F_9/2_ → ^6^H_11/2_ transitions, respectively, but the most intense emission line is in the yellow domain at 579 nm for both CNGG:Dy and CLNGG:Dy crystals. Under the host excitations of λ_ex_ = 272 and 280 nm (Figure 8), it can be seen that the emission lines of Dy^3+^ ions have low intensity suggesting that the energy transfer from the host to Dy^3+^ ions is weak in both investigated crystals. The value of the emission cross-section for yellow emission at 579 nm was calculated to be *σ_em_* = 0.33×10^−20^ cm^2^, being similar in both CNGG:Dy and CLNGG:Dy crystals. Moreover, other laser parameters such as branching ratios *(β_JJ’_)* and Y/B (yellow/blue) ratio, required for efficient lasing in the yellow domain, were determined to have similar or even higher values compared to other crystals (Table 8). Therefore, CNGG:Dy and CLNGG:Dy crystals are promising yellow gain media.

The partial energy levels and the multiplet barycenter (Bc) of Dy^3+^ ions in the CNGG:Dy and CLNGG:Dy crystals were determined by using the absorption and emission spectra at 10 K [23]. The energy levels involved in the yellow emission transition of Dy^3+^ compared with those of the Dy:YAG crystal [73] are given in Table 9.

**Figure 9:**
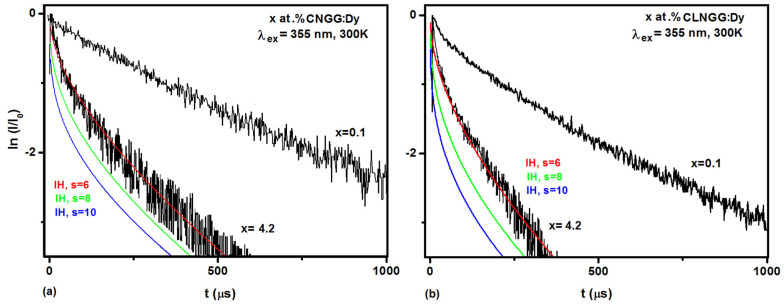
Emission kinetics of the ^4^F_9/2_ level of Dy^3+^ ions in CNGG:Dy (**a**) and CLNGG:Dy (**b**) samples. (Reprinted with permission from Ref. [23], copyright 2018, Elsevier).

The luminescence decays of the ^4^F_9/2_ level of Dy^3+^ ions in CNGG:Dy and CLNGG:Dy crystals at room temperature were measured by observing the yellow line at 579 nm under the excitation wavelength of 355 nm. To find out the lifetime of isolated Dy^3+^ ions, sintered ceramic samples of CNGG:Dy and CLNGG:Dy doped with 0.1 at.% Dy were also measured. The normalized luminescence decays are shown in Figure 9. The decay curve is exponential for the low concentration of 0.1 at.% Dy in the CNGG:Dy ceramic, with a measured lifetime of *τ_0_* ~ 543 μs (Figure 9a), while in the case of the CLNGG:Dy ceramic, the decay has a non-exponential profile (Figure 9b) indicating the presence of ET processes at the doping concentration of 0.1 at.% Dy. In this case, the average lifetime was determined to be *τ_av_* = 405 μs.

For the 4.2 at.% Dy concentration, the decay curves present a non-exponential shape (Figure 9), and the average luminescence lifetimes were estimated to be τ*_av_*=229 μs and τ*_av_* = 143 μs for the CNGG:Dy and CLNGG:Dy crystals, respectively. Using the IH model, the non-exponential decay curves were evaluated, and the experimental transfer functions were determined. From the fitting of the ^4^F_9/2_ luminescence decay, the critical distance (*R_0_*), microparameter of donor-acceptor interaction (*C_DA_*), transfer rates *(W_DA_),* energy transfer rate (*W_ET_*), and quantum efficiency (*ɳ*) were determined (Table 10) and compared with other Dy^3+^-doped crystals in Table 10 [57,58,59]. The analysis of the decay curves highlighted the efficient ET between Dy^3+^ ions in the CNGG:Dy and CLNGG:Dy crystals. The high values of the quantum efficiency of the ^4^F_9/2_ level, *ɳ* ~ 73 %, coupled with the favorable emission cross-sections at 579 nm, *σ_em_* = 0.33 × 10^−20^ cm^2^, indicate that Dy^3+^ ions doped in CNGG:Dy and CLNGG:Dy are potential laser crystals in yellow at 579 nm.

- Pr^3+^ ions

**Figure 10:**
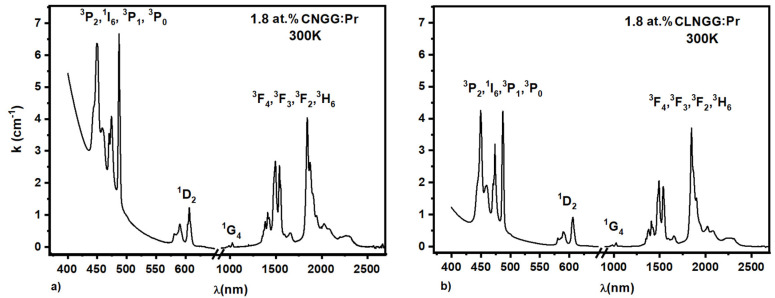
Absorption spectra of Pr^3+^ ions in CNGG:Pr (**a**) and CLNGG:Pr (**b**) crystals. (Reprinted with permission from Ref. [24], copyright 2019, Elsevier).

The absorption spectra of the 1.8 at.% Pr^3+^ ions in CNGG:Pr and CLNGG:Pr crystals (Figure 10) were measured in the 400–2750 nm domain at room temperature [24] and analyzed within the JO theory [48,49]. For the estimation of JO parameters, seven absorption bands of Pr^3+^ ions were considered. Generally, the traditional Judd-Ofelt theory supposes that the 4*f*5*d* configurations are situated at higher energies than the 4*f*^n^ configuration. For the Pr^3+^ ions, the difference between the 4*f*^2^ configuration and 4*f*^1^5*d*^1^ level is only about Δ*E*~15000 cm^−1^, implying the existence of significant mixing between these two configurations. Therefore, a large difference between the calculated and theoretical results is usually obtained, particularly when the ^3^H_4_→^3^P_2_ transition is considered in the JO parameters calculation [74]. These incompatibilities are generated by the theoretical characteristics of the standard JO model and can be eliminated by removing the ^3^H_4_→^3^P_2_ transition or by employing a modified JO analysis.

The modified OJ theory proposed by *Kornienko* et al. [74] assumes that the transition probabilities from the ground level to the 4*f*^1^5*d*^1^ state are higher than those calculated by standard JO theory, and therefore the energy difference between the 4*f*^2^ and 4*f*^1^5*d*^1^ configurations is taken into account, and the absorption oscillator strength is expressed as:

(5) $$S_{\mathrm{mod}}S_{\mathrm{standard}}\times \left[1+\frac{E_{i}-2E_{f}^{0}}{E_{5d}-E_{f}^{0}}\right]$$

where *S_standard_* is the standard oscillator strength and *E_i_*, *E*_5*d*_, and $E_{f}^{0}$ are the energies of the final state, the 4*f*^1^5*d*^1^ state, and the mean energy of the 4*f*^2^ configuration, respectively. For our crystals, the mean energy of the 4*f*^2^ configuration was determined to be $E_{f}^{0}$ = 10578 cm^−1^ for the CNGG:Pr crystal and $E_{f}^{0}$ = 10660 cm^−1^ for the CLNGG:Pr crystal. Based on the absorption and excitation spectra, the energy of the 4*f*^1^5*d*^1^ lower level of the 4*f*5*d* configuration was found to be *E_5d_* = 31348 cm^−1^ (319 nm) in both CNGG:Pr and CLNGG:Pr crystals. Consequently, the modified JO analysis excluding the ^3^H_4_ → ^3^P_2_ + ^1^I_6_ transitions was applied, and the obtained *Ω_t mod_ (t = 2, 4, 6)* intensity parameters are given in Table 11. The values of spontaneous emission probabilities *(A_JJ’_)*, branching ratios *(β_JJ’_)*, and radiative lifetimes (*τ_r_*) for the excited levels of Pr^3+^ ions in CNGG:Pr and CLNGG:Pr crystals [24] were determined based on the values of *Ω_t mod_* parameters, and are also shown in Table 11.

**Figure 11:**
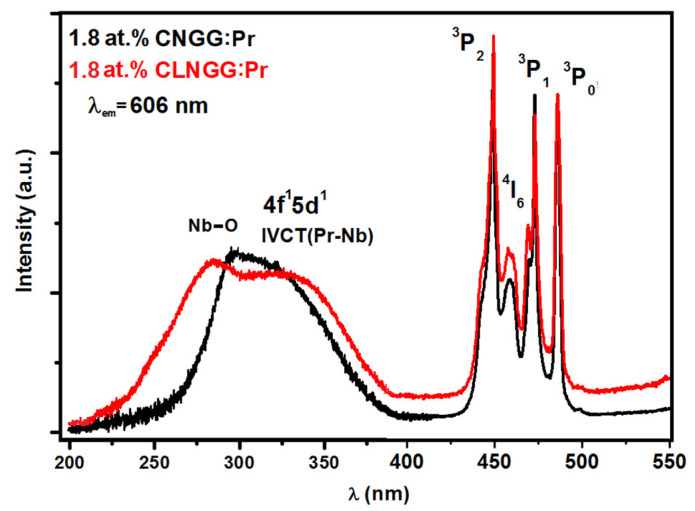
Excitation spectra of Pr^3+^ ions in CNGG:Pr and CLNGG:Pr crystals. (Reprinted with permission from Ref. [24], copyright 2019, Elsevier).

The excitation spectra of the 1.8 at.% Pr^3+^ ions in CNGG:Pr and CLNGG:Pr crystals were recorded by observing the emission line at *λ_em_* = 606 nm assigned to the ^1^D_2_→^3^H_4_ transition (Figure 11). The obtained spectra present a wide UV band in the 200–400 nm spectral range and a group of narrow lines in the 420–490 nm spectral domain assigned to the 4*f*^2^ electronic configuration of Pr^3+^ ions. The wide bands in the UV domain contain two types lines: the first one is associated with the CT bands assigned to the Nb^5+^−O^2−^ interactions into the [NbO_4_] tetrahedrons of the host lattices (peaks at 272 nm for CLNGG and 280 nm for CNGG), while the second type are the peaks placed at λ = 319 nm and attributed to the 4*f*^1^5*d*^1^ lower level of the 4*f*5*d* configuration of Pr^3+^ ions.

However, when the Pr^3+^ and Nb^5+^ ions are both contained in a material, a photo-induced redox process Pr^3+^+Nb^5+^→Pr^4+^+ Nb^4+^ takes place, leading to the formation of an intervalence charge transfer (IVCT) band. If the IVCT band is located at low energies, it can interact with the ^3^P_0,1,2_ manifolds of the Pr^3+^ ions leading to the emission quenching of these manifolds. The quenching of emission from the ^3^P_0_ level in materials containing M^4+^ or M^5+^ metal ions (M = Ti, V, Nb, and Ta) was intensively studied [24,75,76,77,78]. When the IVCT band is positioned at higher energies, there is no influence on the ^3^P_0_ level emissions and intense emissions from the ^3^P_0_ level can be obtained, especially a strong emission in the blue domain attributed to the ^3^P_0_→^3^H_4_ transition [24,77]. Therefore, the UV line situated around 319 nm can contain both the 4*f*^1^5*d*^1^ lower level of 4*f*5*d* configuration and the IVCT band.

**Figure 12:**
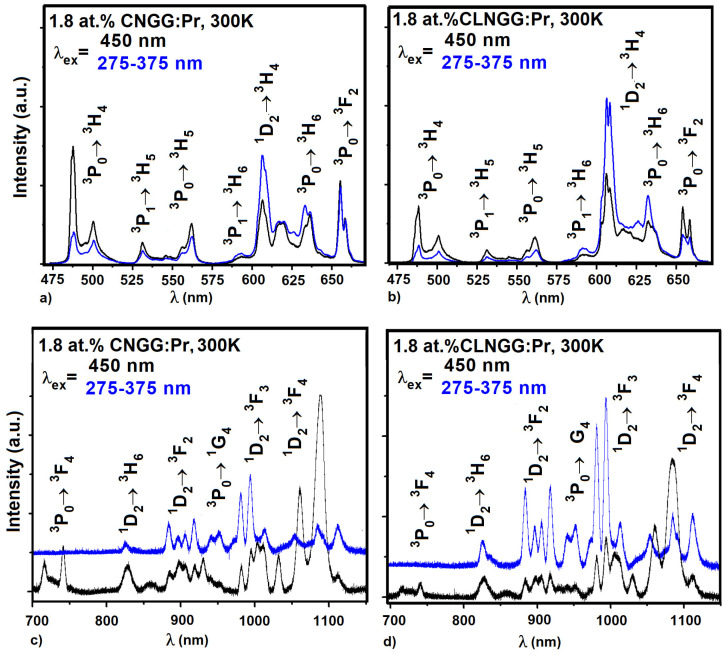
Emission spectra of Pr^3+^ ions in CNGG:Pr and CLNGG:Pr crystals. (Reprinted with permission from Ref. [24], copyright 2019, Elsevier).

The emission spectra of Pr^3+^ ions in CNGG:Pr and CLNGG:Pr crystals in the VIS (Figure 12a,b) and near-infrared spectral ranges (Figure 12c,d) were registered under UV (275–375 nm) and 450 nm excitation wavelengths at room temperature [24]. Under UV excitation, the spectra exhibit very low intensities for the emission lines arising from the ^3^P*_J (J=0,1,2,)_* manifolds, while the emission lines arising from the ^1^D_2_ manifold are intense and well-structured in both spectral domains. Under UV excitation, the electrons migrate from the (4*f*^1^5*d*^1^ level + IVCT) band passing through the ^3^P*_J_* _(*J* = 0,1,2,)_ manifolds to the ^1^D_2_ multiplet, thus leading to obtaining a dominating ^1^D_2_→^3^H_4_ emission [79]. A total quenching of the ^3^P_0_ level emission was observed for the NaYTiO_4_:Pr phosphor [80], where the energy gap between the ^3^P_0_ level and the IVCT band is less than 7400 cm^−1^. For the CNGG:Pr and CLNGG:Pr crystals [24], this energy gap is around 11827 cm^−1^, thus explaining the partial quenching of emission from the ^3^P_0_ level. Under direct excitation at 450 nm in the ^3^P_2_ level, the obtained spectra show more intense emission lines from both ^3^P*_J (J = 0,1,2,)_* and ^1^D_2_ manifolds compared with those obtained under 275–375 nm UV excitation.

Table 12 shows the stimulated emission cross-sections corresponding to the ^3^P_0_→^3^H_4_, ^1^D_2_→^3^H_4_, and ^3^P_0_→^3^F_2_ transitions obtained for the investigated crystals in comparison with other similar crystals. The high values of the emission cross-sections indicate that the CNGG:Pr and CLNGG:Pr are potential laser crystals in the blue, red, and orange domains.

The partial energy level scheme and the manifold barycenter (*B_c_*) of Pr^3+^ ions are presented in detail in Reference [24]. The levels involved in the blue, orange, and red emissions assigned to the ^3^P_0_ → ^3^H_4_, ^1^D_2_ → ^3^H_4_, and ^3^P_0_ → ^3^F_2_ transitions, respectively, of the Pr^3+^ ions in CNGG:Pr and CLNGG:Pr are given and compared with those of the Pr: YAG crystal [84] in Table 13.

**Figure 13:**
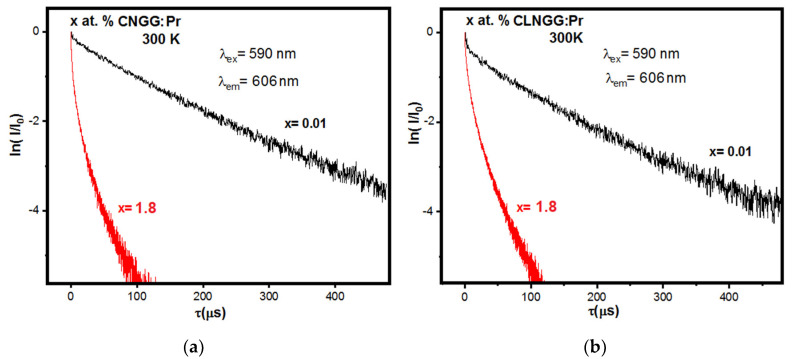
Emission kinetics of the ^1^D_2_ level of Pr^3+^ ions in CNGG:Pr (**a**) and CLNGG:Pr (**b**) samples. (Reprinted with permission from Ref. [24], copyright 2019, Elsevier).

The emission kinetics of ^1^D_2_ level of Pr^3+^ ions in CNGG:Pr and CLNGG:Pr crystals were measured at room temperature by observing the emission line at 606 nm under direct excitation at 590 nm [24]. To obtain the lifetime of isolated Pr^3+^ ions, ceramic samples of CNGG:Pr and CLNGG:Pr doped with 0.01 at.% Pr were also sintered and measured. The normalized luminescence decays are presented in Figure 13. The luminescence decay is exponential for the low concentration of 0.01 at.% Pr in the CNGG:Pr ceramic, with a measured lifetime of τ_0_ ~ 128 μs (Figure 13a), while in the case of the CLNGG:Pr ceramic, the decay has a non-exponential profile (Figure 13b) indicating the presence of ET processes at the doping concentration of 0.01 at.% Pr. In this case, the average lifetime was determined to be τ_av_ = 110 μs. For the doping concentration of 1.8 at.% Pr, the decay curves have a non-exponential shape, and the measured lifetime decrease drastically to *τ_av_* = 18 μs and *τ_av_* = 15 μs, respectively. The quenching emission of the ^1^D_2_ level at room temperature can increase due to the increase of the Pr^3+^-Pr^3+^ non-radiative interactions if the resonance between levels is fulfilled or the ET process is phonon-assisted. Due to the presence of Pr^3+^ multicenters, the application of the IH model does not seem appropriate since the analysis of the ^1^D_2_ decay profiles may lead to inaccurate results.

## 4. Conclusions

The growth and spectroscopic characteristics of RE^3+^ ions (RE = Sm, Dy, Pr) doped in partially disordered Ca_3_Nb_1.6875_Ga_3.1875_O_12_ – CNGG and Ca_3_Li_0.275_Nb_1.775_Ga_2.95_O_12_ – CLNGG crystals were reviewed. High-quality CNGG:RE and CLNGG:RE crystals were grown using the Czochralski crystal growth technique. The JO model was employed to determine the *Ω_t_* (*t = 2, 4, 6*) intensity parameters and to evaluate the spectroscopic properties and laser emission features of the Pr^3+^, Sm^3+^, and Dy^3+^ ions, respectively, doped in the grown crystals. The partial energy levels of each dopant ion were determined. The luminescence decays of the ^4^G_5/2_ (Sm^3+^) and ^4^F_9/2_ (Dy^3+^) levels were evaluated within the IH theory, and the ET parameters were determined. For the Pr^3+^ ion, the emission decays of the ^1^D_2_ level show a drastic decrease of the lifetime values at high doping concentrations due to the cross-relaxation processes, and the application of the IH model does not seem appropriate because may lead to inaccurate results. The high values obtained for the stimulated emission cross-sections indicate that CNGG and CLNGG crystals doped with Sm^3+^, Dy^3+^, and Pr^3+^ ions could be promising materials to achieve laser emission in the orange (Sm^3+^), yellow (Dy^3+^), and blue, orange, and red (Pr^3+^) domains.

## Figures and Tables

**Table 1:** Elemental compositions and lattice parameters of CNGG:RE and CLNGG:RE (RE = Sm, Dy, Pr) grown crystals. (Reprinted with permission from Ref. [47], copyright 2018, Elsevier).

Crystal	Elemental Composition	Lattice Parameters (Å)
CNGG	Ca_3_Nb_1.6875_Ga_3.1875_O_12_	12.508(2) [17]
CNGG:Sm	(Ca_0.966_Sm_0.034_)_3_Nb_1.688_Ga_3.187_O_12_	12.502(5)
CNGG:Dy	(Ca_0.958_Dy_0.042_)_3_Nb_1.686_Ga_3.189_O_12_	12.493(1)
CNGG:Pr	(Ca_0.982_Pr_0.018_)_3_Nb_1.684_Ga_3.191_O_12_	12.525(1)
CLNGG	Ca_3_Li_0.275_Nb_1.775_Ga_2.95_O_12_	12.511(0) [17]
CLNGG:Sm	(Ca_0.966_Sm_0.034_)_3_Li_0.279_Nb_1.777_Ga_2.944_O_12_	12.506(4)
CLNGG:Dy	(Ca_0.958_Dy_0.042_)_3_Li_0.277_Nb_1.776_Ga_2.947_O_12_	12.501(0)
CLNGG:Pr	(Ca_0.982_Pr_0.018_)_3_Li_0.276_Nb_1.775_Ga_2.949_O_12_	12.517(1)

**Table 2:** JO intensity parameters for Sm^3+^ in different oxide crystals with partially disordered structure (Reprinted with permission from Ref. [21], copyright 2017, Elsevier).

Crystal	*Ω*_2_ × 10^−20^ (cm^2^)	*Ω*_4_ × 10^−20^ (cm^2^)	*Ω*_6_ × 10^−20^ (cm^2^)
YAG:Sm [57]	1.2	2.08	1.72
CNGG:Sm	3.06	3.89	2.6
CLNGG:Sm	4.19	4.4	2.49
CaNb_2_O_6_:Sm [58]	6.33	6.49	3.72
LiNbO_3_:Sm [59]	2.11	4.5	1.45
LiNbO_3_:Mg:Sm [60]	1.86	2.82	1.19
LiNbO_3_:Zn:Sm [60]	1.68	2.72	1.12

**Table 3:** Spontaneous emission probabilities (*A_JJ’_)*, branching ratios (*β_JJ’_*), and radiative lifetimes (*τ_r_*) of Sm^3+^ ions in CNGG:Sm and CLNGG:Sm crystals. (Reprinted with permission from Ref. [21], copyright 2017, Elsevier).

Transition from ^4^G_5/2_ →	CNGG:Sm					CLNGG:Sm				
	$\bar{\lambda }$ (nm)	$A_{}^{ED}$ (S^−1^)	$A_{}^{MD}$ (S^−1^)	*β_JJ’_* (%)	*τ_r_*(ms)	$\bar{\lambda }$ (nm)	$A_{}^{ED}$ (S^−1^)	$A_{}^{MD}$ (S^−1^)	*β_JJ’_*(%)	*τ_r_*(ms)
^6^F_11/2_	1359	0.58		0.0009		1361	0.56		0.0007	
^6^F_9/2_	1146	3.21		0.005		1147	4.22		0.006	
^6^F_7/2_	1016	5.24	2.15	0.01		1016	5.92	2.14	0.011	
^6^F_5/2_	936	23.6	6.8	0.04		936	25.84	6.84	0.053	
^6^H_15/2_	880	0.86		0.02		879	0.8		0.02	
^6^F_3/2_	878	4.45	10.76	0.0004		878	5.9	10.7	0.001	
^6^F_1/2_	869	4.05		0.006		869	5.4		0.007	
^6^H_13/2_	782	10.7		0.016		771	11		0.015	
^6^H_11/2_	708	52.2		0.08		708	56.2		0.08	
^6^H_9/2_	647	205		0.32		647	215.8		0.32	
^6^H_7/2_	600	233	25.2	0.41		600	240.8	25	0.37	
^6^H_5/2_	566	14.3	30.2	0.07	1.58	566	17.5	29.97	0.06	1.5

**Table 4:** Emission cross-sections of Sm^3+^ ions in CNGG:Sm and CLNGG:Sm crystals compared to other similar crystals. (Reprinted with permission from Ref. [21], copyright 2017, Elsevier).

Crystals	Transition from ^4^G_5/2_ →	$\bar{\lambda }$	*σ_em_* ×10^−21^ cm^2^
CNGG:Sm	^6^H_5/2_	567	0.187
	^6^H_7/2_	615	0.87
	^6^H_9/2_	662	0.63
CLNGG:Sm	^6^H_5/2_	567	0.184
	^6^H_7/2_	615	1
	^6^H_9/2_	663	0.79
CaNb_2_O_6_:Sm [58]	^6^H_7/2_	610	2.42
LiNbO_3_:Sm [59]	^6^H_7/2_	617	0.82
GdVO_4_:Sm [64]	^6^H_7/2_	604	0.9

**Table 5:** Partial energy levels of Sm^3+^ ions involved in the main emission lines in CNGG:Sm and CLNGG:Sm crystals.

Multiplets	CNGG:Sm [21]	B_c_ (cm^−1^)	CLNGG:Sm [21]	B_c_ (cm^−1^)	YAG:Sm [57]	B_c_ (cm^−1^)
^4^G_5/2_	17630	17852	17633	17853	17601	17784
	17908		17909		17867	
	18846		18850		17883	
^6^H_9/2_	2543	2404	2555	2419	2618	2461
	2491		2525		2568	
	2408		2417		2466	
	2339		2352		2401	
	2238		2244		2254	
^6^H_7/2_	1362	1214	1368	1219	1419	1261
	1289		1299		1371	
	1190		1191		1242	
	1016		1019		1012	
^6^H_5/2_	228	109	233	113	251	130
	98		106		141	
	0		0		0	

**Table 6:** Emission kinetic parameters of the ^4^G_5/2_ level of Sm^3+^ ions in CNGG:Sm and CLNGG:Sm crystals.

IH Fitting	CNGG:Sm [21]	CLNGG:Sm [21]	YAP:Sm [65]	KZnLa(PO)_4_:Sm [66]	LGSO:Sm [67]
S	6	6	6	6	6
R_0_ (Å)	6.59	6.86	7.22	10.96	7.9
C_DA_ (m^6^s^−1^)	5.31 × 10^−53^	7.01 × 10^−53^	5.9 × 10^−53^	4.04 × 10^−52^	3.64 × 10^−52^
W_DA_ (s^−1^)	525	691	416.5	233.08	1497.4
W_ET_ (s^−1^)	325	358	510	865	450
τ_r_ (ms)	1.58	1.5	2.4	4.3	1.89
τ_0_ (ms)(0.1 at.%)	1.483	1.44			
τ_av_ (ms)	1	0.95	2.14	0.911	1.74
ɳ (%)	67	66	89	21	92

**Table 7:** Intensity parameters *Ω_t_* (*t = 2, 4, 6*) and other spectroscopic parameters of Dy^3+^ ions in CNGG:Dy and CLNGG:Dy crystals.

CNGG:Dy [23]							CLNGG:Dy [23]				
Transition from	Level	$\bar{\lambda }$ (nm)	n	$A_{}^{ED}$ (S^−1^)	$A_{}^{MD}$ (S^−1^)	β_JJ’_(%)	$\bar{\lambda }$ (nm)	n	$A_{}^{ED}$ (S^−1^)	$A_{}^{MD}$ (S^−1^)	β_JJ’_(%)
^4^F_9/2_	^6^F_1/2_	1335	1.9039	0.14		0	1315	1.8997	0.15		0
	^6^F_3/2_	1280.5	1.9054	0.1064		0	1280	1.9007	0.1054		0
	^6^F_5/2_	1152.8	1.9101	11.2		0.0061	1160	1.9050	10.78		0.006
	^6^F_7/2_	999.8	1.9183	6.8	9.84	0.009	999.2	1.9136	6.75	9.78	0.0092
	^6^H_5/2_	930.9	1.9235	5.3		0.0029	931.7	1.9187	5.27		0.0029
	^6^F_9/2_	849.4	1.9315	12.4	9.66	0.012	849.6	1.9267	12.33	9.58	0.012
	^6^H_7/2_	829.6	1.9338	12.6	5.99	0.01	829.1	1.9337	12.64	6.008	0.0104
	^6^F_11/2_	765.1	1.9427	32.3	90.8	0.06	764.6	1.9381	32.15	89.14	0.0678
	^6^H_9/2_	747.2	1.9457	26.4	5.52	0.01	747.2	1.9410	26.18	5.49	0.0177
	^6^H_11/2_	661.5	1.9637	107.8	21.8	0.07	660.9	1.9591	107.2	21.73	0.072
	^6^H_13/2_	574.9	1.9921	1131.8		0.628	574.8	1.9874	1122.7		0.627
	^6^H_15/2_	480.8	2.0468	311.2		0.17	480.8	2.0410	308.57		0.172
	A_T_(^4^F_9/2_) = 1801.6 s^−1^, *τ_r_* = 0.55 ms						A_T_(^4^F_9/2_) = 1786.5 s^−1^, *τ_r_* = 0.559 ms				
*Ω*_t_	*Ω*_2_= 5.04 × 10^−20^ cm^2^ *Ω_4_*= 1.81 × 10^−20^ cm^2^ *Ω_6_* = 1.53 × 10^−20^ cm^2^						*Ω*_2_= 5.29 × 10^−20^ cm^2^ *Ω_4_*= 1.49 × 10^−20^ cm^2^ *Ω_6_* = 1.37 × 10^−20^ cm^2^				
*χ*	1.18						1.08				

**Table 8:** Emission cross sections (*σ_em_*), branching ratios *(β_JJ’_)*, and Y/B (yellow/blue) ratio of the yellow transition of Dy^3+^ ions in CNGG:Dy and CLNGG:Dy compared with other materials. (Reprinted with permission from Ref. [23], copyright 2018, Elsevier).

Crystals	^4^F_9/2_ →^6^H_13/2_ *σ_em_* (×10^−20^ cm^2^)	Y/B	*ß_calc_*(%)	*τ_rad_*(ms)	*τ_exp_*(ms)
CNGG:Dy [23]	0.337	2.95	62.8	0.55	0.438
CLNGG:Dy [23]	0.338	3	62.7	0.559	0.332
YAG:Dy [69]	0.3	1.3	50.96	0.9	0.669
Gd_2_SiO_5_:Dy [70]	0.68	1.66	54.35	0.542	0.497
GdVO_4_:Dy [71]	0.9	4	68.3	0.264	0.084
GGG:Dy [72]	0.26	1.32	49	1.1	0.79

**Table 9:** Partial energy levels of Dy^3+^ ions involved in ^4^F_9/2_ → ^6^H_13/2_ yellow laser transition in the CNGG:Dy and CLNGG:Dy crystals.

Multiple’s	CNGG:Dy [23]	Bc (cm^−1^)	CLNGG:Dy [23]	Bc (cm^−1^)	YAG:Dy [73]	Bc (cm^−1^)
^4^F_9/2_	21,547	21,169	21,554	21,172	21,551	21,096
	21,210		21,210		21,119	
	21,136		21,140		21,056	
	21,001		21,001		20,896	
	20,950		20,954		20,859	
^6^H_13/2_	3960	3776	3960	3777	3948	3731
	3936		3938		3821	
	3845		3847		3776	
	3774		3776		3720	
	3697		3695		3699	
	3643		3647		3590	
	3575		3577		3564	

**Table 10:** Emission kinetic parameters of the ^4^F_9/2_ manifold of Dy^3+^ ions in CNGG:Dy and CLNGG:Dy crystals.

IH Fitting	CNGG:Dy [23]	CLNGG:Dy [23]	Bi_4_Si_3_O_12_:Dy [57]	LSO:Dy [58]	Lu_2_SiO_5_:Dy [59]
s	6	6	6	6	6
R_0_ (Å)	11.8	12.413	9.15	8.90	8.9
C_DA_ (m^6^s^−1^)	0.512 × 10^−52^	0.908 × 10^−52^	0.65 × 10^−51^	7.8 × 10^−52^	5.3 × 10^−52^
W_DA_ (s^−1^)	1843	2481	1107.6	1569.4	1066
W_ET_ (s^−1^)	681	681	856	338	390
τ_r_ (ms)	0.550	0.559	1.684	0.635	0.635
τ_0_ (ms)(0.1 at.%)	0.543	0.405			
τ_av_ (ms)	0.229	0.143	0.690	0.202	0.509
ɳ (%)	73	72	41	32	80

**Table 11:** Spontaneous emission probabilities (*A_JJ’_*), branching ratios (*β_JJ’_*), and radiative lifetimes (*τ_r_*) of the ^3^P_0_ and ^1^D_2_ excited levels of Pr^3+^ ions in CNGG:Pr and CLNGG:Pr crystals.

Multiplets		CNGG:Pr [24]				CLNGG:Pr [24]			
		*ν (cm^−1^)*	*A^ED^*	*A^MD^*	*β*	*ν (cm^−1^)*	*A^ED^*	*A^MD^*	*β*
^3^P_0_	^1^D_2_	3721	9.31		0.0002	3716	11.63		0.0002
	^1^G_4_	10,680	909.7		0.02	10,572	1086		0.02
	^3^F_4_	13,475	3503		0.07	13,406	4457		0.08
	^3^F_3_	13,970	0		0	13,962	0		0
	^3^F_2_	15,229	14,644		0.32	15,225	18,298		0.33
	^3^H_6_	16,046	1495		0.032	16,142	1320		0.02
	^3^H_5_	17,999	0		0	18,024	0		0
	^3^H_4_	20,298	23,725		0.52	20,303	29,254		0.53
		Ʃ A = 44,286 s^−1^, *τ_r_* = 22 μs				Ʃ A = 54,426.63 s^−1^, *τ_r_* = 18.3 μs			
^1^D_2_	^1^G_4_	6959	429		0.09	6856	501.6		0.09
	^3^F_4_	9754	1998.4		0.45	9690	24,44.6		0.43
	^3^F_3_	10,249	187.7	11.2	0.04	10,246	233	14	0.04
	^3^F_2_	11,508	574.6	5.4	0.12	11,509	709.3	6.67	0.13
	^3^H_6_	12,325	514		0.11	12,426	644		0.11
	^3^H_5_	14,278	26		0.05	14,308	319		0.05
	^3^H_4_	16,577	714		0.15	16,587	751		0.13
		Ʃ A= 4460 s^−1^, *τ_r_* = 224 μs				Ʃ A= 5623.2 s^−1^, *τ_r_* = 177.8 μs			
*Ω_t mod_*	*Ω*_2_ = 3.607 × 10^−20^ cm^2^ *Ω*_4_ = 3.580 × 10^−20^ cm^2^ *Ω*_6_ = 1.264 × 10^−20^ cm^2^ *rms = 0.086 × 10^−20^ cm^2^*					*Ω*_2_ = 4.563 × 10^−20^ cm^2^ *Ω*_4_ = 4.451 × 10^−20^ cm^2^ *Ω*_6_ = 1.105 × 10^−20^ cm^2^ *rms = 0.12 × 10^−20^ cm^2^*			

**Table 12:** Calculated emission cross-sections (*σ_em_*) of Pr^3+^ ions in CNGG:Pr and CLNGG:Pr in comparison with other Pr-doped crystals. (Reprinted with permission from Ref. [24], copyright 2019, Elsevier).

Crystal	^3^P_0_→^3^H_4_ *σ_em_* (cm^2^)	^1^D_2_→^3^H_4_ *σ_em_* (cm^2^)	^3^P_0_→^3^F_2_ *σ_em_* (cm^2^)
CNGG:Pr	5.72 × 10^−20^	0.48 × 10^−20^	1.9 × 10^−19^
CLNGG:Pr	7.48 × 10^−20^	0.51 × 10^−20^	2.96 × 10^−19^
CaSGG:Pr [81]	7.3 × 10^−20^	-	1.02 × 10^−19^
SrLaGa_3_O_7_:Pr [82]	3.43 × 10^−20^	-	0.44 × 10^−19^
GGG:Pr [83]	-	-	1.24 × 10^−19^

**Table 13:** Energy levels of Pr^3+^ ions involved in the ^3^P_0_ → ^3^H_4_, ^1^D_2_ → ^3^H_4_, and ^3^P_0_ → ^3^F_2_ emission transitions in CNGG:Pr and CLNGG:Pr crystals.

Pr^3+^ Multiplets	CNGG:Pr [24]	B_c_ (cm^−1^)	CLNGG:Pr [24]	B_c_(cm^−1^)	YAG:Pr [84]	B_c_(cm^−1^)
^3^P_0_	20,603	20,603	20,601	20,601	20,534	0
^1^D_2_	17,235 17,105 16,974 16,590 16,512	16,883	17,227 17,111 16,978 16,575 16,534	16,885	17,210 17,088 16,881 16,409 16,400	16,798
^3^F_2_	5464 5409 5368 5335 5303	5375	5479 5410 5352 5332 5311	5376	6994 6973 6943 6876 6857	6929
^3^H_4_	- 623 554 517 - - 66 21 0	-	- 625 555 513 - - 74 22 0	-	- 742 576 533 - - 50 19 0	-

## Data Availability

Not applicable.
